# State Work with Infectious Diseases of Animals1Presented at the annual meeting of the State Agricultural Society, at St. Paul, January 10 and 11, 1900.

**Published:** 1900-03

**Authors:** M. H. Reynolds

**Affiliations:** St. Anthony Park, Minn.


### STATE WORK WITH INFECTIOUS DISEASES OF ANIMALS.[1]

By M. H. Reynolds, M.D., V.M.
ST. ANTHONY PARK, MINN.

Mr. President and Gentlemen of the State Agricultural Society: There are certain things which I wish to accomplish in the presentation of this paper. I wish to interest all members of the State Agricultural Society and the Minnesota Stock Breeders' Association in the work of the State Board of Health, and particularly in the work of the veterinary department. It is your work, and done mainly for your business interests. Mistakes in this work may cost your interests hundreds of thousands of dollars. Good work may save as much. The success of the veterinary sanitation depends on the support of stockmen and veterinarians. It is important that those most interested should be informed concerning the work to be done and methods of accomplishing it.

The Minnesota State Board of Health consists of nine members, secretary and general executive officer, bacteriological laboratory, and veterinary department.

Minnesota is credited with having one of the best if not the best State law relating to infectious diseases of domestic animals. There are certain points in this law which I wish to present in order that our work may be well understood.

[1] Presented at the annual meeting of the State Agricultural Society, at St. Paul, January 10 and 11, 1900.

Section 1, Chapter 233, gives authority to the State and various local boards to take such steps as they may deem necessary to control infectious diseases among domestic animals. These boards are given authority to regulate or prohibit the arrival in or departure from this State and the towns, villages, and cities thereof of exposed or infected animals.

Section 2 reads as follows :

"Any person who knows of, or has reason to suspect, the existence of any contagious or infectious disease in any domestic animal, shall forthwith give notice thereof to the local board of health of the town, village, or city where such animal is kept. Within twenty-four (24) hours after any local board of health shall have received notice that any domestic animal is infected with any such disease, or has been exposed thereto, it shall give notice thereof in writing to the State Board of Health."

Section 4 provides that no animal shall be killed by any of these boards until it has been adjudged to be affected with a contagious or infectious disease, either by a physician or veterinary surgeon properly selected and employed. The State Board is given authority to kill any animal which has been exposed, although not at that time affected with the disease, if in the judgment of this board it is necessary to do so.

Section 5 provides for the owner's protection, giving him authority under certain conditions to protect, demand post-mortem by three experts, and the privilege of appraisal if the animal is found to be free from infectious diseases.

Section 7 provides that local boards must carry out and enforce orders and regulations of the State Board. It also gives the State Board authority to require two or more boards to act together when desirable.

Section 9 provides penalties. Suit may be brought in a justice's court. Fine may not be less than $25 nor more than $100, or imprisonment not less than thirty nor more than ninety days. This section also provides that any member of the local board of health neglecting or refusing to carry into effect the provisions of this law or the rules or regulations made by the State Board of Health shall be guilty of a misdemeanor punishable by a fine of not less than $25 nor more than $100, and each day's neglect or refusal constitutes a separate or independent misdemeanor.

Section 11 authorizes private owners whose stocks are not affected to establish private quarantine in self-defense during the prevalence of an infectious disease among domestic animals, and makes such private quarantine legal. Permit me to explain at this point, as illustrating my thought, that members of this and similar associations should be personally interested and informed concerning this work. Section 11 was suggested by the Secretary of the Stock Breeders' Association, and I consider this one of the best points in the new law.

Section 1, Chapter 47, reads as follows :

"It shall be the duty of the owner, or of any other person having in charge any swine that have died of any disease, immediately upon the fact of such death by disease coming to his knowledge, to bury the same at least three (3) feet below the surface of the ground, or burn the same so that the carcass is consumed. No person shall sell, give away, or offer for sale any swine that have died of any disease or have been killed on account of any disease. No person shall convey upon or along any public highway, or other public ground, or any private land, except his own, any diseased swine, or swine that have died of or have been slaughtered on account of any disease. It shall be unlawful for any person negligently or willfully to allow his hogs or those under his control afflicted with any disease to escape his control or run at large."

Then follows a definition of penalties.

[Glanders discussion omitted because already covered in the paper on "State Control of Glanders," by the same author, and recently published in this JOURNAL.]

(To be continued.)

---

McKillip Veterinary College is conducting a practitioner's course at which some fourteen of the profession are attending.

Five graduates in veterinary medicine are attending the regular course at McKillip Veterinary College.

Western Veterinary College has had some thirty-three students enrolled during the present college year. This school expects during the coming year to construct a building especially for the purpose of college work and instruction.

Dr. James S. Catanach, of New York City, intends that, so far as he can set the example, there shall be a greater need of horses, appearing among his clients regularly now with three horses abreast, or rather two horses, and a mule gracing the centre place.

Veterinarians officiating at the September Horse Show at Bryn Mawr, Pa., were Drs. C. J. Marshall, C. T. Goentner, and Alex. Glass.

